# Criterion Validity of the Problematic Khat Use Screening Tool (PKUST-17) in Ethiopia

**DOI:** 10.1192/bjo.2025.10852

**Published:** 2025-10-08

**Authors:** Awoke Mihretu, Solomon Teferra, Yohannes Gebreegziabhere, Kassahun Habtamu, Abebaw Fekadu

**Affiliations:** Department of Psychiatry, WHO Collaborating Center for Mental Health Research and Capacity Building, College of Health Sciences, Addis Ababa University, Addis Ababa, Ethiopia; Department of Nursing, College of Health Sciences, Debre Berhan University, Debre Berhan, Ethiopia; School of Psychology, College of Education and Behavioral Studies, Addis Ababa University, Addis Ababa, Ethiopia; Center for Innovative Drug Development and Therapeutic Trials for Africa (CDT-Africa), Addis Ababa University, Addis Ababa, Ethiopia; Global Health and Infection Department, Brighton and Sussex Medical School, Brighton, UK

**Keywords:** Amphetamine-type stimulants (ATS), khat use disorder, screening tool, criterion validity, Ethiopia

## Abstract

**Background:**

The use of amphetamine-type stimulants such as khat has been spreading quickly in eastern parts of Africa, the Arabian region and Asia. However, screening for the provision of early intervention has been inadequate, primarily because of the lack of culturally acceptable and valid screening tools.

**Aims:**

To evaluate the the accuracy of the Problematic Khat Use Screening Tool (PKUST-17) in screening for khat use disorder against the DSM-5 criteria for substance use disorder.

**Method:**

A cross-sectional validation study was conducted in Ethiopia from February to December 2018, among a randomly selected sample of 506 individuals. The study subsample comprised 236 participants. We used the DSM-5 criteria for stimulant use disorders as the standard for determining the criterion validity and optimal cut-off score for the PKUST-17, using the receiver operating characteristic (ROC) curve. The DSM-5 criteria for substance use disorders were examined by researchers in a subsample of 232 participants. The PKUST-17 uses a five-point Likert scale (0–4), with total scores ranging from 0 to 68. At the optimal cut-off scores, sensitivity and specificity were determined. In addition, we conducted multivariate logistic regression analysis to evaluate potential convergent validity of the tool.

**Results:**

The area under the ROC curve showed good performance of the PKUST-17 (0.78, 95% CI 0.70–0.85, *P* < 0.001). A cut-off score above 17 demonstrated a sensitivity of 72% and specificity of 73%. The positive predictive value was 77.6% and the negative predictive value was 65.8% in identifying stimulant use disorder, as per the DSM-5 criteria. Among others, problematic khat use was significantly associated with higher World Health Organization Disability Assessment Schedule 2.0 scores (adjusted odds ratio 1.78, 95% CI 1.04–3.03, *P* < 0.01) and more depressive symptoms (adjusted odds ratio 4.10, 95% CI 2.36–7.12, *P* < 0.05).

**Conclusions:**

We found that the PKUST-17 is a valid tool for screening for khat use disorder against the DSM-5 criteria for substance use disorder, and identifying high-risk problematic khat users.

Globally, amphetamine-type stimulants are the second most common illicit drugs, after cannabis.^1,2^ Khat is an amphetamine-type stimulant (with main components of cathinone and cathine) and it increases dopamine, serotonin and norepinephrine in the central nervous system.^3^ Khat use is highly prevalent in East Africa and the Arabian Peninsula, particularly in Somalia, Kenya, Ethiopia and Yemen, and among immigrants from these regions in Europe and North America.^4^ In Ethiopia, the prevalence of khat use disorder ranges from 20 to 74.6%.^5^ A study conducted among Yemeni residents in the UK, as well as residents of Yemen and Saudi Arabia, found that 51, 48.9 and 52.2% of khat chewers, respectively, reported khat use disorder as defined by khat psychological dependence.^6–8^ Many people chew khat for functional and social reasons, whereas others use it for self-treatment from distress associated with continued use or withdrawal.^9,10^ Khat consumption has long been socially regulated;^9^ recently, however, problematic khat use has become a significant concern. Khat use disorder is found to be a valid construct, with many of the criteria resembling those in the DSM-5 for substance use disorders.^11^ Individuals who meet the criteria for problematic khat use report consuming larger amounts, experiencing cravings, being unable to quit and facing negative consequences, including mental health issues, sexual dysfunction, physical health problems, and social and financial difficulties.^9^

Hence, screening and early intervention of problematic khat use are crucial. However, the lack of a valid, easy-to-administer and culturally appropriate screening tool that can differentiate individuals at different risk levels hinders healthcare providers’ ability to screen, implement brief interventions and refer individuals to specialised care.^12,13^

Previous measures of problematic khat use are of poor quality, inadequately addressing criterion validity. For example, the study by Gebrehanna et al^5^ overlooked dependence and focused on harmful use. Additionally, it did not employ a proper standard to evaluate the criterion validity of its assessment tools. The study used frequency of use (twice or more per week) as a ‘gold standard’ measure of problematic khat use.^5^ However, another study identified the duration of chewing sessions (≥6 h) as a more significant predictor of khat dependence than frequency of use.^7^ Another proposed measure for problematic khat use disorder is the Severity of Dependence Scale (SDS), which primarily assesses psychological dependence, such as impaired control. The internal reliability of the SDS-khat was good, with a Cronbach’s alpha coefficient of 0.76, and test–retest reliability of 0.93. Moreover, the proposed SDS-khat showed a significant correlation with self-reported reasons for khat chewing.^14^ However, the SDS captures only a limited aspect of problematic khat use (i.e. only psychological).

Previous validation studies of khat use screening tools based their assumption on alcohol and cannabis use studies (i.e. frequency, duration and harms). Methodological studies indicated that applying indicators from other constructs, such as alcohol and cannabis use, to another construct such as khat use may lack domain specificity and contextual relevance.^15^ Therefore, there is a need for the development and validation of a screening tool for problematic khat use based on a bottom-up approach. We started by conceptualising problematic khat use in the setting and developed the Problematic Khat Use Screening Tool (PKUST-17), using rigorous procedures, including systematic reviews, qualitative studies, cognitive interviews, expert workshops and pilot studies.^16^ The PKUST-17 has demonstrated acceptable content, semantic, construct and structural validity, making it useful in research and clinical practice.^16^ However, its application for screening has been limited because of the absence of cut-off scores, which would help to differentiate individuals with khat use disorder from those with casual use. To address this critical gap, we conducted this study, which aimed to establish a cut-off score for the PKUST-17 against the DSM-5 criteria for stimulant use disorder. This study will be significant in helping to facilitate the early screening, intervention and referral of cases with problematic khat use.

## Method

### Study setting, design and period

The study was conducted in the Gurage zone, Wolkitie town and Kebena district, located 158 km south of Addis Ababa, the capital of Ethiopia. The study setting allowed the inclusion of both urban and rural residents. Details about the study setting have been reported in a previous study.^9^ This study is a validation cross-sectional study. The data were collected from 16 August 2018 to 11 December 2018.

### Participants, sample size determination and sampling

To determine the minimum sample size needed for the study, a sample size determination formula for the study of the sensitivity of a screening tool was used.^17^ The anticipated sensitivity and specificity were 75 and 80%, respectively.^5^ We also assumed an alpha level of 0.05, a desired precision of 0.1 and a 74% prevalence of the problem (i.e. problematic khat use).^11^ Thus, the minimum sample size required to determine the criterion validity and establish a cut-off score for the PKUST-17 was 236. In addition to the scientific rationale for determining a sample size sufficiently powered to achieve our desired outcomes, we also had logistical reasons for limiting the sample size to a specified number. The costs associated with hiring Master’s level mental health professionals were high and exceeded the project’s budget. Hence, 236 participants were randomly subsampled from the larger validation study (*N* = 506) we conducted previously. Participants aged 18–65 years and living in the study area (Gurage zone, central Ethiopia) for 2 years were eligible for the study. Kish’s method of sampling was used to select participants.^18^ Using the sampling frame from subdistrict health posts, we performed two-stage random selection. First, households were randomly selected from the list of eligible households. Then, a random person was selected from each household and interviewed from the Kish grid.

### Data collection procedure

Trained lay data collectors conducted the interviews to gather information about demographic characteristics, khat use history and other relevant variables, followed by administering the PKUST-17. Master’s level trained mental health professionals interviewed each participant against the DSM-5 criteria for stimulant use disorder to diagnose khat use disorder. All interviews were conducted in community settings. To minimise information bias, the order of administering the PKUST-17 and DSM-5 was randomised. Participants were interviewed using either the PKUST-17 or DSM-5 first, with the order determined randomly.

### Measures

#### PKUST-17

The PKUST-17 consists of 17 items rated on a five-point Likert scale from lowest response value ‘0’ to ‘4’.^19^ The total score ranges from 0 to 68. The tool reflects a unidimensional model with excellent internal consistency (*α* = 0.93). Item response theory analysis showed that each item effectively distinguishes participants across the spectrum of problematic khat use, and the tool functions best among participants with moderate problematic khat use. Latent profile analysis (LPA) identified three latent classes with varying levels of severity. The detailed development process and psychometric properties of the PKUST-17 were reported in our previous studies.^16,19^

#### DSM-5 criteria for stimulant use disorders

We used the DSM-5 criteria for stimulant use disorder as the standard to determine the optimal cut-off score for the PKUST-17.^20^ A diagnosis of khat use disorder is made when an individual meets two to three symptoms for mild, four to five symptoms for moderate, or six or more symptoms for severe, as outlined in the DSM-5. A previous study in Ethiopia found that the DSM-5 had an acceptable construct validity to measure khat use disorder.^21^

#### Sociodemographic characteristics and pattern of khat use

A structured questionnaire was used to collect data on sociodemographic characteristics, such as age, gender and marital status, and pattern of khat use, such as amount of khat, frequency of khat use, duration and time of khat use session.

#### Depressive symptoms

The nine-item Patient Health Questionnaire (PHQ-9) was used to screen depressive symptoms. The PHQ-9 has been adapted and validated in Ethiopia and has an acceptable psychometric properties. It is a unidimensional scale with a cut-off score of 5 and above in rural settings.^22^

#### Disability

The 12-item World Health Organization Disability Assessment Schedule (WHODAS 2.0) was used to assess functional impairment as a result of problematic khat use. The WHODAS 2.0 measures six domains of functional impairment, including community participation, life activities, self-care, getting along with people and mobility. The instrument has been adapted and validated in Ethiopia among people with severe mental disorders,^23^ with acceptable psychometric properties, including content, convergent, concurrent and structural validity.

#### Social support

The three-item Oslo Social Support Scale (OSSS-3) was used to measure perceived social support.^24^ The total score ranges from 3 to 14; 3–8 indicates poor social support, 9–11 indicates moderate social support and 12–14 indicates strong social support.

#### Stressful life events

We used the List of Threatening Events (LTE) to assess the presence of stressful life events in the past 30 days. The LTE is a list of significant and threatening events such as loss of relationships, death of close persons and being in jail. LTE items are dichotomous with a ‘yes’ or ‘no’ response format. The LTE questionnaire has been frequently used in rural Ethiopia.^25^

#### Alcohol use disorder

The Alcohol Use Disorder Identification Test (AUDIT) was used to assess the pattern of alcohol use. AUDIT was developed by the World Health Organization to screen individuals for alcohol use disorder.^26^ It has ten items assessing alcohol consumption behaviour in the past year. AUDIT items are polytomous, with total scores ranging from 0 to 40. The cut-off for problematic alcohol use is eight or more. In Ethiopia, the AUDIT has been frequently used and reported to have a very high internal consistency (Cronbach’s *α* = 0.84).^27^

### Data collection

The lay data collectors had at least a diploma-level education and more than 5 years in data collection. They underwent 16 h of comprehensive training on participant engagement, interview techniques for closed-ended items, response recording and ethical considerations in data collection. Master’s-level mental health clinicians diagnosed khat use disorder by using the DSM-5 criteria for stimulant use disorder. They were highly experienced and had been working at substance use disorder rehabilitation centres in Ethiopia for over 2 years. They also received a brief orientation on the study’s objectives and data collection procedures. All interviews were conducted in community settings. To minimise bias, the order of administering the PKUST-17 and DSM-5 was randomised. Participants were interviewed using either the PKUST-17 or DSM-5 first, with the order determined randomly. This randomisation helps reduce information bias from the participants and prevents the interviewers’ temptation to conform to the results of the initially administered tool. The principal investigator closely supervised the data collection process and randomly attended interviews, ensuring completeness and addressing any gaps. After the data were collected, we conducted double data entry using EpiData as an additional quality control mechanism.^28^

### Data analysis

The criterion validity of the PKUST-17 against the DSM-5 criteria for stimulant use disorder was evaluated using the receiver operating characteristic (ROC) curve. We used Youden’s index to know the accuracy of our tool. Youden’s index ranges from 0 to 1, with values closer to 1 indicating better performance.^29^ The area under the curve (AUC) is used to know the overall ability of PKUST-17 to discriminate between those with and without problematic khat use. An AUC value of 1.0 indicates perfect discrimination, 0.5 indicates discrimination no better than chance, greater than 0.8 indicates good discrimination and less than 0.7 indicates poor discrimination.^30^ We did a multivariate logistic regression analysis to test the trustworthiness of the cut-off for problematic khat use. We tested the association between the hypothesised predictor variable, such as the WHODAS 2.0, OSSS-3, PHQ-9, LTE, pattern of khat use and sociodemographic variables, and levels of problematic khat use, categorised as below and above the cut-off threshold. Stata 16 for Windows and MedCalc 2023 for Windows (MedCalc Software Ltd, Ostend, Belgium; see https://www.medcalc.org) were used for data analysis. We used the 30-item Standards for Reporting Diagnostic Accuracy Studies (STARD) checklist) to improve the quality of reporting of diagnostic accuracy studies.^31^ Before conducting the analysis, we removed four observations by using list-wise deletion, as this was unlikely to significantly bias our results. Since the missing values were deemed negligible and had no major impact on the main analysis, we retained them.

### Ethics approval and consent to participate

The authors declare that all procedures contributing to this work comply with the ethical standards of the relevant national and institutional committees on human experimentation and with the Helsinki Declaration of 1975, as revised in 2013. The study was conducted after obtaining ethical approval from the Institutional Review Board of the College of Health Sciences, Addis Ababa University (ref 008/18/psy). We sought and obtained informed consent from participants. Written consent was obtained from all participants. Non-literate participants gave fingerprints to signify their willingness to participate, and this was witnessed by an independent person. We ensured that participants made a free and informed consent to participate in the study by providing a clear information sheet in Amharic, containing all necessary information about potential harms and benefits, purpose of the study, privacy and voluntary participation. The study maintained privacy and confidentiality during data collection, handling and reporting. Participants at high risk of problematic khat use were given contact information for health facilities addressing substance use problems and general information about addiction.

## Results

### Sociodemographic characteristics

A total of 506 current khat chewers were interviewed for the overarching aim of the project, and a subsample of 232 were interviewed for the current criterion validation study. The detailed characteristics of the participants are summarised in Table 1.

**Table 1:** Sociodemographic characteristics of the study participants

	The whole sample (*N* = 506)	Criterion validation subsample (*n* = 232)
Variables	Percentage	Percentage
Age in years, mean (s.d.)	34.4 (±13)	35.75 (±11.51)
Gender, % male	90	90
Religion, %		
Orthodox	31	34.60
Muslim	59	49.3
Protestant	5	11.30
Catholic	5	4.80
Marital status, %		
Single	40	28.17
Married	54	66.78
Divorced or widowed	6	5.05
Education, %		
No formal education	24	27.74
Primary education	28	24.66
Secondary education	25	23.97
College and above	23	23.63
Occupation, %		
Farmer	14	18.21
Merchant	19	24.40
Student	6	6.19
Employee	23	25.42
Unemployed	38	25.78
Relative wealth, % identified themselves as low	42	58.90
Residence, % rural	41	75.68

### Criterion validity

#### Criterion validity of the PKUST against mild DSM-5 stimulant use disorder

**Fig. 1:**
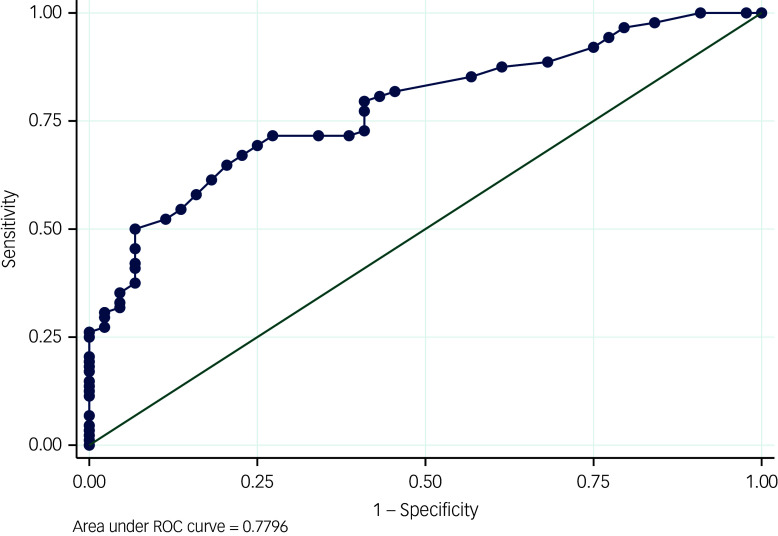
Receiver operating curve (ROC) for mild Problematic Khat Use Screening Tool (PKUST-17) against DSM-5.

The prevalence of mild problematic khat use disorder according to the PKUST-17 (cut-off score of >17) was 57%, with a sensitivity of 71.6%, specificity of 72.7% and an AUC of 0.78 (95% CI 0.70–0.85, *P* < 0.001) (Fig. 1). At this cut-off score, the positive predictive value (PPV) was 77.6% and the negative predictive value (NPV) was 65.8%. Youden’s J-value for the PKUST-17 was 0.45.

#### Criterion validity of the PKUST against moderate DSM-5 stimulant use disorder

**Fig. 2:**
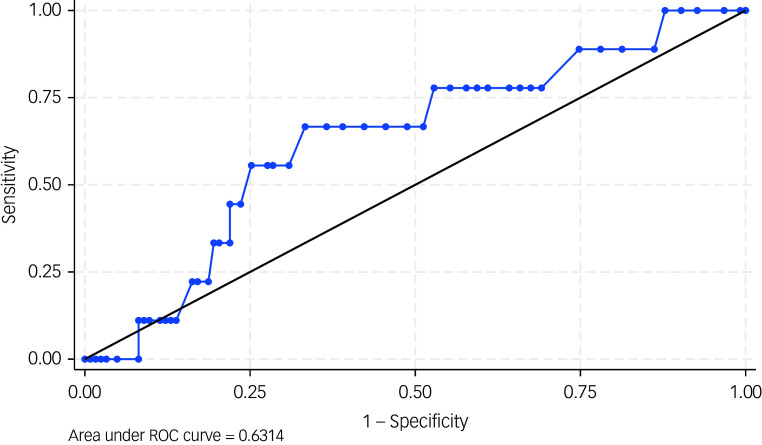
Receiver operating curve (ROC) for the Problematic Khat Use Screening Tool (PKUST-17) against moderate DSM-5 stimulant use disorder.

The prevalence of moderate problematic khat use disorder according to the PKUST-17 (cut-off score of >27) was 38.6%, with a sensitivity of 66.7%, specificity of 63.4% and an AUC of 0.63 (95% CI 0.64–0.81, *P* < 0.001) (Fig. 2). The PPV was 53.4% and the NPV was 75.2%. Youden’s J-value for the PKUST-17 was 0.3.

#### Criterion validity of the PKUST against severe DSM-5 stimulant use disorder

Overall, the prevalence of severe problematic khat use disorder according to the PKUST-17 (cut-off score of >20) was 55.3%, with a sensitivity of 66%, specificity of 69.5% and an AUC of 0.71 (95% CI 0.63–0.79, *P* < 0.001) (Fig. 2). The PPV was 72.7% and the NPV was 62.4%. Youden’s J-value for the PKUST-17 was 0.35.

### Convergent validity

In the binary logistic regression analysis, before controlling for confounders, some variables were found to be statistically associated with a high risk of problematic khat use. These variables included younger age (odds ratio 0.98, 95% CI 0.96–0.99), being male (odds ratio 2.63, 95% CI 1.41–5.00), being married (odds ratio 0.54, 95% CI 0.37–0.78), higher WHODAS 2.0 scores (odds ratio 2.11, 95% CI 1.43–3.12), more symptoms of depression (odds ratio 5.63, 95% CI 3.59–8.81), using khat for self-treatment of distress (odds ratio 2.56, 95% CI 1.33–4.93), chewing khat daily (odds ratio 5.81, 95% CI 3.95–8.55), chewing khat in the morning (odds ratio 6.41, 95% CI 3.83–10.75), experiencing more stressful life events (odds ratio 1.98, 95% CI 1.39–2.83) and having strong social support (odds ratio 0.24, 95% CI 0.14–0.42).

After adjusting for potential confounders, we found that the odds of those with depressive symptoms having problematic khat use were four times greater compared with those without depression (adjusted odds ratio 4.10, 95% CI 2.36–7.12). As the WHODAS 2.0 score increased by one point, the odds of having problematic khat use increased by twofold (adjusted odds ratio 1.78, 95% CI 1.04–3.03). Daily chewing and chewing in the morning emerged as the strongest predictor of problematic khat use, with individuals who chew khat daily and in the morning six and four times more likely to have problematic khat use compared with their counterparts, respectively (daily chewing: adjusted odds ratio 6.38, 95% CI 3.89–10.46; chewing in the morning: adjusted odds ratio 4.07, 95% CI 2.14–7.73). Finally, being male had four times higher odds of problematic khat use compared with being female (adjusted odds ratio 4.35, 95% CI 1.59–12.50) (Table 2).

**Table 2:** Multivariant logistic regression analysis of factors associated with problematic khat use using the cut-off score of the Problematic Khat Use Screening Tool (PKUST-17)

Variables	Not problematic khat users	Problematic khat users	Crude odds ratio [95% CI]	Adjusted odds ratio [95% CI]
Age in years, mean	35.79	33.11	0.98 [0.96–0.99]*	0.96 [0.94–0.99]*
Gender, *n* (%)				
Female	15 (6.22)	37 (14.62)	1	1
Male	216 (85.38)	230 (93.88)	2.63 [1.41–5.00]*	4.35 [1.59–12.50]*
Marital status, *n* (%)				
Single	83 (42.35)	113 (57.65)	1	1
Married	155 (57.62)	114 (42.38)	0.54 [0.37–0.78]*	0.97 [0.54–1.76]
Divorced and widowed	15 (45.45)	18 (54.55)	0.88 [0.42–1.85]	1.11 [0.33–3.76]
WHODAS 2.0 scores, mean	3.5	7.7	2.11 [1.43–3.12]*	1.78 [1.04–3.03]*
Depressive symptoms, *n* (%)				
No	221 (62.08)	135 (37.92)	1	1
Yes	32 (22.53)	110 (77.47)	5.63 [3.59–8.81]*	4.10 [2.36–7.12]*
Reason for khat use, *n* (%)				
Functional and social reasons	194 (57.57)	143 (42.43)	1	1
For self-treatment from distress	79 (42.47)	107 (57.53)	2.56 [1.33–4.93]*	0.90 [0.37–2.19]
Manner of chewing, *n* (%)				
With others	164 (49.7)	166 (50.3)	1	1
Alone	90 (54.5)	75 (45.4)	0.87 [0.60–1.25]	0.94 [0.57–1.56]
Frequency of chewing, *n* (%)				
Not daily	195 (72.8)	73 (27.2)	1	1
Daily	78 (30.6)	177 (69.4)	5.81 [3.95–8.55]*	6.38 [3.89–10.46]*
Morning chewing habit, *n* (%)				
No	252 (61.5)	158 (38.5)	1	1
Yes	20 (18)	91 (82)	6.41 [3.83–10.75]*	4.07 [2.14–7.73]*
Problematic alcohol use, *n* (%)				
No	18 (69.2)	8 (30.8)	1	1
Yes	46 (41.4)	65 (58.6)	2.13 [0.90–5.04]	1.81 [0.61–5.38]
Stressful life events, *n* (%)				
No	142 (59.7)	113 (42.8)	1	1
Yes	96 (40.3)	96 (57.2)	1.98 [1.39–2.83]*	1.22 [0.72–2.05]
Social support level, *n* (%)				
Poor	27 (33)	55 (67)	1	1
Moderate	105 (44.7)	130 (55)	0.61 [0.36–1.03]	1.23 [0.60–2.54]
Strong	121 (66.8)	60 (33)	0.24 [0.14–0.42]*	0.48 [0.22–1.00]

WHODAS 2.0, World Health Organization Disability Assessment Schedule. *P < 0.05.

After adjusting for potential confounders, we found that the reasons for use, chewing alone or with others, levels of social support, stressful life events and problematic alcohol use were not found to be statistically significantly associated with the risk of problematic khat use (*P* > 0.05 for all variables).

## Discussion

The aim of the study was to evaluate the accuracy of the PKUST-17 in diagnosing khat use disorder against DSM-5 criteria for stimulant use disorder. We established cut-off scores for the PKUST-17 with acceptable sensitivity and specificity. The cut-off we established is able to identify factors previously reported to be associated with problematic khat, suggesting convergent validity of the scale and the usefulness of the cut-off. The findings show that the PKUT-17 is likely to be a very useful measure for screening, intervention and referring individuals with problematic khat use.^16^

The PKUST-17 is found to have acceptable criterion validity, suggesting its usefulness in identifying those with problematic khat use. The ROC curve showed moderate accuracy of the PKUST in identifying problematic khat use (AUC = 0.78). The sensitivity and specificity of the PKUST-17 are also not different from other problematic substance use instruments. For example, a systematic review of the psychometric properties of other problematic substance use tools indicates that sensitivity ranges from 54 to 97%, and specificity from 50 to 96%.^32^ Sensitivity for the AUDIT ranges from 51 to 97%, and specificity from 78 to 96%.^33^ As khat use is deeply embedded in the culture of the study setting, many individuals may screen positive for problematic khat use.^10^ This cultural factor might also contribute to the PKUST-17 identifying many individuals as positive with its current psychometric properties. Previous studies have also reported a high proportion of problematic khat users when using the DSM-5 criteria for stimulant use disorders, which is comparable to the current study. This further supports the screening power of the PKUST-17.^11^

The results indicate that measuring the severity of problematic khat use based on the number of diagnostic criteria outlined in the DSM-5 guidelines may have limited utility. Therefore, clinicians and researchers should consider alternative measures for assessing problematic khat use severity. This could include developing a revised version of the PKUST-17, revising the diagnostic threshold of the DSM-5 or identifying a different standard that more accurately classifies problematic khat use.

The normative perception of khat consumption may be influenced by cultural factors, such as its widespread use in the region, or by limitations related to the small sample size of the current study. These factors may have made it more difficult for the assessment to accurately identify true moderate and severe cases.^34^ Additionally, a high prevalence of the condition within the test population can affect the predictive values, which may indirectly lead to a lower AUC and sensitivity/specificity profile for the PKUST-17.^34^ Khat is widely used for recreational purposes and is generally socially accepted, which may contribute to its use not being recognised as a medical or psychological concern. The present study further supports this notion by identifying a positive association between social support and high-risk khat use.^10^ The current study adds to a growing body of literature supporting the use of valid and brief screening tools for identifying substance misuse, and builds upon prior research demonstrating the validation of different screening tools for different substances both in primary care and among the general population.^35–37^ The results of the current study found that the PKUST-17 screening tool is a valid and potentially useful instrument among the general population to screen high-risk problematic khat users. The results suggest that measuring the severity of problematic khat use by using the number of diagnostic criteria, per DSM-5 guidelines, may have limited utility. Clinicians and researchers might consider alternative measures of problematic khat use, such as developing a new version of the PKUST-17 or identifying a different standard when focusing on the severity of problematic khat use.

Participants with higher risk of problematic khat use group tend to chew khat daily and in the morning. They also had higher functional impairment and depressive symptom scores. Previous studies reported that patterns of use, such as frequency of khat use and morning chewing of khat, are important indicators of problematic khat use.^9,11,13^ Similar findings to the current study have also been reported, showing that problematic khat users are likely to have common mental disorders, depression symptoms, functional impairment and poor quality of life.^11^ The significant association we observed between the dichotomised problematic khat use using the cut-off of the PKUST-17, and such variables as depressive symptoms, WHODAS 2.0 scores and daily khat use, add further evidence for the construct validity of the PKUST-17.

Initiatives such as screening, brief intervention and referral to treatment for problematic substance use^38^ can only be effectively implemented with reliable, valid, simple and brief screening tools. Tools such as the PKUST-17 will have an irreplaceable role. It is, therefore, essential to provide training and awareness creation workshops for healthcare workers and other professionals in various settings, both clinical and non-clinical, about the use of the PKUST-17 for the screening, brief intervention and referral to treatment of potential cases.

Using a screening tool and giving feedback to individuals about their substance use behaviour, including khat use, is considered to be a brief intervention in itself.^39^ Prevention interventions can raise awareness of the problem and induce changes. This new tool is likely to increase motivation and behaviour change, similarly to other tools.^39^ Individuals with khat use who could be screened negative can reinforce that what they are doing is responsible and encourage them to continue their current low-risk khat use patterns. This benefits khat users’ well-being and the healthcare system by reducing healthcare costs arising from early screening.

### Strengths and limitations

This study is novel in establishing a cut-off for the PKUST-17, a psychometrically sound, comprehensive and user-friendly tool for assessing problematic khat use. The criterion validity and cut-off of the PKUST-17 were cross-checked by examining the association with hypothesised related factors, providing evidence for both the criterion and convergent validity of the measure. However, the present study has several limitations. For instance, its cross-sectional nature precludes the assessment of the predictive validity of the PKUST-17. Furthermore, causality might not be established about the factors reported to be associated with problematic khat use. In addition, we used the DSM-5 criteria for substance use disorder as a gold standard. However, the construct substance use disorder in the DSM-5 is developed based on a Western-based conceptualisation of substance use disorder. Although the DSM-5, a tool primarily developed within a North American cultural context, appears capable of capturing problematic khat use, the local PKUST-17 tool offers a more comprehensive set of criteria for identifying such use. Another limitation of the study is the potential for response biases; however, we hypothesise that these biases will be random for both the PKUST-17 and DSM-5 tools, and will have minimal influence on the study’s results. The use of the PKUST-17 in other settings should be accompanied by proper adaptation and validation to ensure its relevance to the local context. Readers are recommended to consider these limitations when interpreting the findings of this study.

In conclusion, the PKUST-17 has demonstrated criterion validity with a cut-off score of above 17, making it a suitable brief measure for screening high-risk problematic khat users for interventions or research. The PKUST-17 can be valuable for improving the screening of individuals exhibiting khat user behaviour who are experiencing problematic khat use. Implementing education on the risks associated with khat use and improving awareness of available treatment resources could have important policy and public health implications. Such efforts may contribute to early detection of khat use disorder and improve clinical outcomes. Moreover, increased awareness could drive greater demand for intervention services, support the establishment of rehabilitation centres and encourage the training and deployment of mental health professionals to address khat-related problems more effectively. Future studies should explore the applicability of the PKUST-17 for detecting other types of amphetamine-type stimulants. We also recommend evaluating the tool’s sensitivity to change.

**Fig. 3:**
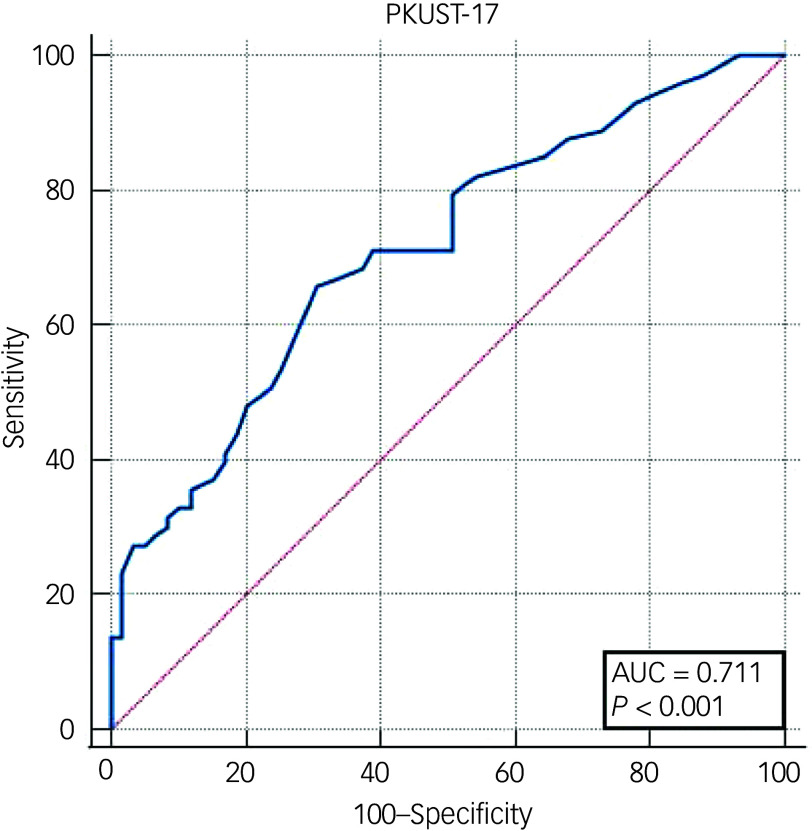
Receiver operating curve (ROC) for the Problematic Khat Use Screening Tool (PKUST-17) against severe DSM-5 stimulant use disorder. AUC, area under the curve.

## Data Availability

The data that support the findings of this study are available from the corresponding author, A.M., on reasonable request.
